# Editorial: Screening and discovering novel biological biomarkers by omic-data to revolutionize tumor management

**DOI:** 10.3389/fgene.2025.1611885

**Published:** 2025-06-27

**Authors:** Binglin Wang, Yinan Jiang, Peijie Wu, Enrique Medina-Acosta, Zhongyi Yan

**Affiliations:** ^1^ School of Basic Medical Sciences, Henan University, Zhengzhou, China; ^2^ Children’s Hospital of Pittsburgh, School of Medicine, University of Pittsburgh, Pittsburgh, PA, United States; ^3^ College of Basic Medical Sciences, Chengdu University of Traditional Chinese Medicine, Chengdu, China; ^4^ Laboratory of Biotechnology, Center for Bioscience and Biotechnology, State University of North Fluminense, Campos dos Goytacazes, RJ, Brazil

**Keywords:** biomarkers, multi-omics data, therapeutic targets, immunotherapy, cancer

Cancer remains a formidable global health challenge, characterized by molecular heterogeneity and adaptive mechanisms that drive tumor progression, metastasis, and therapeutic resistance (Bray et al., 2024). High-throughput technologies have revolutionized cancer research, enabling systematic biomarker discovery through multi-omics analyses (Goodwin et al., 2016). For example, single-cell RNA sequencing (scRNA-Seq) and spatial transcriptomics have enabled the resolution of tumor heterogeneity at subcellular levels (Longo et al., 2021;Gulati et al., 2025). These innovations underscore the shift from bulk tissue analysis to spatially and temporally resolved molecular profiling, which is critical for deciphering dynamic tumor-immune interactions (Hsieh et al., 2022). The integration of multi-omics data has accelerated the identification of biomarkers with clinical utility. Below, we contextualize the studies in this Research Topic within recent breakthroughs in oncology.

Multi-omics integration allows a comprehensive view of the mechanisms underlying any disease. Ahmed et al. characterized genetic and epigenetic changes in AML progression using Omni-C, ATAC-seq, and RNA-seq data. Differential interaction analysis showed significant 3D chromatin landscape reorganization between relapse and diagnosis samples (Ahmed et al., 2024). Uveal melanoma (UVM) is the most common primary intraocular malignancy. However, treatment outcomes are unsatisfactory, and the long-term prognosis remains dismal. To address this challenge, Zhang et al. employed bioinformatics and identified that two TRP channel-related long noncoding RNAs (TCRLs), AC092535.4 and *LINC01637*, could serve as novel prognostic biomarkers for UVM and may present potential therapeutic targets (Zhang et al., 2024). Using multi-omics data, Zhao et al. found that *FANCI* is significantly upregulated in multiple tumor types. High *FANCI* expression correlates with poor prognosis in specific cancers and is associated with higher immune cell infiltration and tumor mutation burden. *FANCI* emerges as a promising biomarker for cancer prognosis and diagnosis, with potential as a novel therapeutic target (Zhao et al., 2025).

Kinesin family proteins and metabolic reprogramming are emerging as central players in tumor progression (Chen et al., 2024b). The expression of *KIF18B* was correlated with immune infiltration in the tumor microenvironment. *KIF18B* is a key factor affecting the prognosis of glioblastoma (GBM) patients, and its targeting may provide a new therapeutic method for GBM patients (Su et al., 2025). Yang B et al. demonstrated that *KIF18B* correlated with hepatocellular carcinoma progression potentially via activation of the Wnt/β-catenin-signaling pathway, suggesting its broader role in epigenetic regulation (Yang et al., 2020). Targeting metabolic enzymes like *GFPT1* has gained traction. *In vivo*, silencing of *GFPT1* attenuated the immune escape of breast cancer cells by reducing PD-L1 levels (Tang et al., 2024). These findings align with Liang et al. observation that high *GFPT1* level was associated with increased cytoplasmic translation, activation of oncogenic pathways, and infiltration of M2 macrophages, indicating that *GFPT1* may be a novel prognostic biomarker and an indicator of chemotherapy response in invasive breast carcinoma (Liang et al., 2024).

Immune-related signatures are reshaping therapeutic strategies. Ni et al. constructed an 8-gene prognostic model (*AK2*, *CXCL11*, *TYK2*, *ANGPT4*, *IL20RA*, *MET*, *ENPP6*, and *CA12*) in pancreatic ductal adenocarcinoma (PDAC), enhancing prognostic accuracy and potentially therapeutic decision-making in PDAC, offering valuable insights for evaluation in clinical practice (Ni et al., 2024). Wan et al. found that high *MARCKS*, *MACC1*, and *GRB10* expression in their tumors correlated with poorer survival rates in endometrial cancer patients. High expression of *NINJ2* correlated with higher survival rates and higher sensitivity to radiation therapy (Wan et al., 2025).

Immunotherapy based on immune checkpoint inhibitors (ICIs) has become a prominent focus in the development of novel anti-tumor drugs and has been tested in several human clinical trials. Thus, an immune-related gene prognostic index (IRGPI) was developed by Chen et al., providing a systematic analysis of distinct and molecular characteristics in papillary renal cell carcinoma (PRCC) (Chen et al., 2024a).

This Research Topic exemplifies the power of multi-omics approaches in discovering novel biological biomarkers and unraveling the molecular complexity of cancer. By identifying biomarkers that reflect tumor biology, immune interactions, and therapeutic vulnerabilities, these studies pave the way for precision oncology. As we advance, integrating multi-omics data with AI and innovative therapeutics will be pivotal in transforming cancer care, ultimately improving survival and quality of life for patients worldwide.
